# Enhancing the Antifouling Properties of Alumina Nanoporous Membranes by GO/MOF Impregnated Polymer Coatings: In Vitro Studies

**DOI:** 10.3390/jfb15030050

**Published:** 2024-02-20

**Authors:** Mona Moaness, Sara A. M. El-Sayed, Hanan H. Beherei, Mostafa Mabrouk

**Affiliations:** 1 Refractories, Ceramics and Building Materials Department, Advanced Materials, Technology and Mineral Resources Research Institute, National Research Centre, 33 El Bohouth St., Dokki, Cairo P.O. Box 12622, Egypt; drmonabiomaterials@gmail.com (M.M.); sarali_87@yahoo.com (S.A.M.E.-S.); hananh.beherei@gmail.com (H.H.B.); 2 Academy of Scientific Research and Technology (ASRT), Cairo P.O. Box 11516, Egypt

**Keywords:** alumina NPMBs, antifouling, spin coating, graphene oxide, ZIF-8 MOF

## Abstract

Nanoporous membranes (NPMBs) have been the focus of interest of many scientists in the last decade. However, the fouling phenomenon that takes place during the implantation period blocks pores and causes failure in the local implant. In this study, alumina NPMBs were developed using electrochemical anodization through two steps. Furthermore, graphene oxide (GO), free and impregnated with ZIF-8 MOF, was synthesized and loaded in a mixture of PVDF/PVP polymer matrix at different ratios, and was applied to the produced NPMBs using spin-coater. The NPMBs were characterized before and after coating by SEM/EDX, TEM, FTIR, XRD, contact angle and AFM. The antifouling features of the NPMBs were analyzed against two different bacterial species. The prepared alumina NPMBs demonstrated homogeneous porous structures with pore sizes ranging from 36 to 39 nm. The coated layers were proven to possess microporous coatings on the surfaces of the NPMBs. The numbers of released ions (Al and Zn) from the coated NPMBs were below the allowed limits. Bovine serum albumin (BSA) uptake in artificial cerebrospinal fluid (ACSF) was impressively reduced with the presence of coating materials. In addition, the antifouling behavior of the coated NPMBs against the selected strains of bacteria was greatly enhanced compared with the pure alumina NPMBs. Finally, NPMBs’ uncoated and polymer-coated membranes were tested for their ability to deliver donepezil HCl. The results reveal the downregulation of donepezil release, especially from NPMBs coated with PVDF/PVP 0.5GO. It is advised to use the current antifouling materials and techniques to overcome the limitations of the inorganic NPMBs implants.

## 1. Introduction

Applications of controlled drug delivery have significantly increased as a result of recent improvements in biomedical sciences, innovative materials and technology [1,2]. The aims of controlled drug delivery include regulating the drug profile over time for optimal therapeutic advantages and the safe delivery of the required drug dosage to certain areas in the human body. When Folkman et al. [3] discovered that a rabbit can made to pass out by circulating its blood inside a tube exposed to anesthetic gas, this represented the beginning of controlled drug delivery in the 1960s, and this was the first case of implanted medical delivery [4].

Devices that replace a part or function as a portion of it, or become the entire biological structure, are known as implants. Implants are currently used in various sections of the body for a variety of purposes, including drug delivery systems, neural prosthetics, orthopedics, pacemakers, cardiovascular stents, and defibrillators [5,6,7,8,9,10]. Implanted devices are prone to failure despite their obvious therapeutic benefits for a number of reasons, including a lack of tissue integration, infection, inflammation, and, in the worst instance, complete bodily rejection. To enhance the efficacy of the implants, their properties must be developed. Since the development of nanotechnology, there has been an interest in using this innovative science to solve the issue of implant failure. Due to this, new fields of research on drug-coated implants have developed. This is a convergence technology that involves combining at least two different research disciplines to solve a problem [11].

Among the recently developed implanted devices, there has been increasing attention payed to nanoporous membranes in the field of controlled drug delivery. In particular, nanoporous membranes produced by electrochemical anodizing techniques are well suited to the manufacturing of drug reservoirs due to their high porosity and narrow pore size distributions (pore diameters up to 10 nm and stretched across broad regions of a few square centimeters) [12]. Other nanofabrication techniques, such as ultraviolet (UV) or e-beam lithography, are unable to achieve both a high porosity and a narrow pore size distribution. Furthermore, complicated pore morphologies, such as asymmetric or funnel-shaped nanopores, can be easily designed with an adequate control of electrochemical anodizing parameters (voltage, current density, and temperature) [13]. Anodic nanoporous oxides have a high biocompatibility, as well as a chemical and thermal stability, and can be improved further by surface functionalization methods [14].

Nanoporous anodic alumina is a biocompatible material that is electrically insulated, optically clear, and relatively stable chemically. It has been widely used in a variety of implants, such as electronic, optoelectronic, sensing, and dental, as well as orthopedic implants. While studies on alumina nano-template fabrication date back to the 1960s [15], they have become more common in research and manufacturing following the work of Masuda et al. [16]. Because of the development of enhanced anodization procedures, well-defined, ordered nano-architecture templates are possible. Furthermore, nanoporous alumina can be grown directly on the surface of a metal implant [17]. Alumina nanoporous templates have a very large size range, with pore diameters ranging from 5 nm to 50 nm [18] and an array thicknesses exceeding 100 nm [19].

Despite the fact that aluminum oxide is a bio-inert material [20], biocompatibility tests are still required depending on the application. In vitro investigations on immunoisolation applications have indicated no difference between the control group and the porous aluminum oxide capsules [21]. However, aluminum oxide and surface-modified aluminum oxide samples are compared in vivo. Because of the presence of charged crystalline lattice imperfections, the surface of the alumina membrane is charged. These flaws produce a net dipole moment, which causes biological materials to adhere to the surface, resulting in pore clogging and surface fouling. In order to achieve an enhanced performance, there has been an increasing amount of attention paid to modified organic or organic–inorganic composite coats.

Carbonaceous nanomaterials, like CNTs and GOs, have been utilized routinely in the potential coatings of nanoporous membranes. In a typical phase of the inversion method, Ayyaru et al. [22] functionalized GOs with sulfonic acid groups and combined them with polyvinylidene fluoride (PVDF) and polyvinylpyrrolidone (PVP) to create a sulfonated GO (SGO)/PVDF composite of an ultrafiltration membrane. Due to the substitutional sulfonic acid groups with a strong and deep hydration layer, the result was a great improvement in the permeability and anti-fouling properties of the membrane as compared with the pure PVDF and the unaltered GO/PVDF composite membranes.

Researchers have examined the nanocomposite membrane, which is a heterogeneous combination of inorganic nanomaterials as nanofillers and a polymer matrix, to enhance the performance of polymeric membranes and the fouling resistance [23]. It is hypothesized that this combination produces membranes that are more resilient and more hydrophilic. Additionally, it improves the performance, longevity, and fouling resistance of the membrane [24]. TiO_2_, boehmite, silver, and carbon nanomaterials, like carbon nanotubes (CNTs) and graphene oxide (GO), have been applied as nanofillers to improve the performance of membrane coatings made of nanocomposite materials based on PVDF. Due to the PVDF polymer’s excellent thermal stability, chemical resistance, and mechanical strength, it is widely used in industrial applications, particularly in the treatment of water and wastewater. However, the high hydrophobicity of PVDF membranes restricts their uses and leads to significant membrane fouling by proteins, microbes, colloids, and other organic materials, which ultimately promotes the creation of cake layers. Moreover, researchers substituted the GO surfaces with functional groups and nanostructures to enhance the effect of GO on membrane performance [25]. Metal organic frameworks (MOFs) are a nanostructure that is frequently added to GO surfaces [26]. This is a result of the variety of features it possesses, including organized channels and functional groups provided by metal clusters or organic connections [26].

Some of the cognitive and behavioral abnormalities linked to Alzheimer’s disease are likely associated with reduced cholinergic transmission in the central nervous system, according to the widely accepted cholinergic hypothesis. The acetylcholinesterase enzyme, which typically breaks down acetylcholine, is selectively and rapidly inhibited by the donepezil drug, which improves cholinergic transmission and alleviates Alzheimer’s dementia symptoms. Donepezil may also act through inhibiting glutamate-induced excitatory transmission by downregulating NMDA receptors and regulating amyloid proteins, which have been shown to have a notable and considerable impact on Alzheimer’s disease’s process [27].

Accordingly, we prepared alumina nanoporous membranes (NPMBs), using two steps of electrochemical anodization, that could be used as implantable devices for controlled drug delivery. A recent review highlighted the potential of using nanoporous alumina structures as drug carriers for localized medication delivery [28]. We applied a porous coating layer made of PVDF/PVP on the surface of NPMBs using a spin-coater. To overcome the restrictions of PVDF, we added GO and MOF-derived ZnO nanoparticles to the PVDF polymer matrix in the current experiment. In this research, the developed NPMBs, and the coating materials were characterized before and after coating using several characterization techniques, such as SEM/EDX, TEM, FTIR, XRD, contact angle and AFM. Moreover, according to the ion concentrations of the artificial cerebrospinal fluid (ACSF), antifouling tests were performed. The antifouling features were tested against two different bacterial species. Finally, uncoated and polymer-coated alumina nanoporous membranes (NPMBs) were tested for their ability to deliver donepezil locally as a drug model to treat and decrease symptoms of some CNS disorders.

## 2. Materials and Methods

### 2.1. Materials

Aluminum foil was purchased from Kingcheng, Shanghai, China (99.9%), with a thickness of 1.3 mm. Acetone (99.3%) was purchased from Piochem, Gizza, Egypt. sodium hydroxide (Mwt. = 39.997 g/mol) was purchased from El-Nasr pharmaceutical chemicals company, Cairo, Egypt. Absolute ethanol (Mwt. = 46.07 g/mol) was purchased from Merck, Darmstadt, Germany. Perchloric acid (71–73%) was purchased from Advent, Mumbai, India. Oxalic acid dihydrate (Mwt. = 126.07 g/mol) was purchased from Advent, Mumbai, India. Extra pure chromium trioxide (Mwt. = 99.99 g/mol) was purchased from Laboratory Rasayan, Gujarat, India. Orthophosphoric acid (85%) was purchased from El-Nasr pharmaceutical chemicals company, Cairo, Egypt, and sulfuric acid (95–97%) was purchased from Merck, Darmstadt, Germany. The materials used for producing the coating layers were sodium nitrate NaNO_3_ (99%, Mwt. = 84.99 g/mol) purchased from LOBA Chemie, Mumbai, India; sulfuric acid H_2_SO_4_ (95–97%, Mwt. = 98.07 g/mol) purchased from Sigma-Aldrich, Darmstadt, Germany; graphite (C) (Mwt. = 12.01 g/mol) purchased from Sigma-Aldrich, Buchs, Switzerland; and potassium permanganate KMnO_4_ (Mwt. = 158.03 g/mol) and hydrogen peroxide H_2_O_2_ (30%, Mwt. = 34.01 g/mol) purchased from Spectrum, Halton City Texas, USA. 2-methylimidazole (2-MeIM) (99% minimum assay with Mwt. = 82.10 g/mol), zinc nitrate (Zn(NO_3_)_2_ × 6H_2_O) (99% purity), triethylamine (TEA, ≥99.5%) and polyvinyldene difluoride (PVDF) were purchased from Alfa Aesar Thermo Fisher Chemicals, Karlsruhe, Germany, and polyvinyl pyrrolidone (PVP) (Mwt. = 40,000) was purchased from Sigma-Aldrich, Burlington, MA, USA. N, N–dimethyl formamide (DMF) was purchased from Merck, Ganagnam-gu, Seoul, South Korea. Methanol (MeOH, 99.8%, Mwt. = 32.04 g/mol) was purchased from Fisher Chemicals, and was a product of Couva, Trinidad and Tobago; N-hydroxysuccinimide (NHS, 98+%) was purchased from Acros organics, Shanghai, China; 1-ethyl-3-(3-(dimethylamine)propyl)-carbodiimide hydrochloride (EDC, 97%) was purchased from Acros organics, Geel, Belgum.

#### 2.1.1. Preparation and Separation of Alumina NPMBs

For the preparation of alumina NPMBs, a high-purity aluminum sheet was cut into pieces with dimensions of 4.0 cm × 4.5 cm that were subjected to a heat treatment in air at 500 °C for 3 h. After that, a surface treatment of the Al sheets was done by sonication in acetone and a 1 M NaOH solution for 10 min and 3 min, respectively. Then, the Al foils were individually and electrochemically polished in a mixed solution of ethanol and perchloric acid at a 3:1 v/v ratio, with the Al sheet acting as the anode and Pt mesh acting as the cathode. This procedure was carried out with a voltage of 20 V for 3 min in an ice bath under stirring conditions. After electropolishing, the sheets were rinsed thoroughly with ethanol and distilled water. The electrochemical anodization was carried out in 0.3 M oxalic acid at 40 V for 5 h. in an ice bath, followed by etching to remove the formed oxide layer using a 1.8 wt.% H_2_CrO_4_ and 6 wt.% H_3_PO_4_ solution at 70 °C, then the second anodization was carried out under the same conditions as the first for 10 h. For the separation of the AAO membrane from the underlying Al substrate, a third anodization was done in 12 M H_2_SO_4_ at the same voltage as the second for 140 min, followed by immersion in a 1.8 wt.% H_2_CrO_4_ and 6 wt.% H_3_PO_4_ solution at room temperature for 60 min.

#### 2.1.2. Preparation of Graphene Oxide (GO)

Graphene oxide (GO) was prepared according to the following steps: First, we placed in a dry conical flask 5 g of sodium nitrate and 250 mL of sulfuric acid (98%). The previous mixture was homogenized using a magnetic stirrer at 70 °C until the complete dissolving of sodium nitrate (about 30 min of stirring). Then, 5 g of graphite was added to the mixture in the sonicator (hot/boiled water) at 80 °C for 60 min, the mixture was returned to the magnetic stirrer at 70 °C for 45 min, and the stirring was continued till it reached room temperature (black color). The conical flask was put in an ice bath at around 0 °C on the magnetic stirrer and 30 g of potassium permanganate was added, with the addition of potassium permanganate at 2 g every 5 min to avoid an increase in the temperature. Then, stirring was continued for 30 min at 0 °C (the mixture’s color was green). This was followed by sonication for 1 h at room temperature (color became brown) and the stirring was continued at 35 °C overnight. Afterwards, the obtained mixture was sonicated at 80 °C for 30 min (dark brown color), then a hot plate stirrer was used. The temperature was raised to 98 °C with the addition of 460 mL of deionized water and stirring was continued for 1 h (color changed from dark brown to normal brown). Another measure of hot deionized water (1400 mL) and 50 mL of hydrogen peroxide (5% H_2_O_2_) were added, and the temperature was maintained at 98 °C. The heat was switched off and the stirring was continued overnight. After the experiment’s completion, the sample was filtered and washed via centrifugation (Centrifuge condition: 8000 rpm, 15 min and 5 °C) using deionized water four times, and once with ethanol. The collected precipitate was dried at 60 °C overnight.

#### 2.1.3. Preparation of Zinc—MOF/GO (ZIF-8 @ GO) Composites

**Figure 1:**
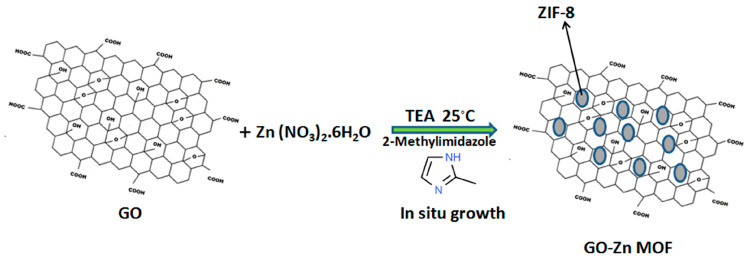
Synthesis of ZIF-8@GO via in situ growth.

The synthesis of ZIF-8/GO hybrid nanosheets was accomplished through the in situ growth of ZIF-8 into GO nanosheets, as shown in Figure 1. To make the ZIF-8/GO hybrid nanosheets, 50 mg of the GO powder was mixed in a 25 mL methanol solution containing 0.1833 g of Zn(NO_3_)_2_x6H_2_O. The solution was then sonicated for 30 min to produce a homogeneous GO suspension solution. This combination was promptly added to a 25 mL solution of TEA containing 3-methylimidazole (3.24 g) and aggressively mixed for 1 h. Finally, the produced ZIF-8/GO hybrid nanosheets were washed with methanol, centrifuged (6000 rpm for 10 min) three times, and dried for 24 h in a 60 °C oven [25].

### 2.2. Coating Materials Preparation

PVDF and PVP were dissolved separately in DMF with weight percentages of 12.5 and 2.5 wt.%, respectively [29]. After the complete dissolving of the two polymers, they were blended together and stirred for 2 h to ensure the homogeneity of the mixture. Moreover, GO and ZIF-8 MOF@GO (250 µg/mL) along with 20 mM and 50 mM of EDC and NHS, respectively, were added; they were then dissolved in DMF to activate the GO suspension to convert the carboxylic acid groups that decorate the GO nanoparticles into amine-reactive esters, then sonicated and stirred for 3 h. The resultant mixture was then added to the PVDF/PVP blend. Then, the polymer blend was transferred to a vacuum spin-coater and 2 mL of the polymer blend was added to the surface of alumina NPMBs using a syringe; then, the apparatus was operated on two runs, each run lasting 1 min with a spinning speed of 200 rpm. The resultant coated membranes were left to dry overnight at room temperature.

#### 2.2.1. Scanning Electron Microscopy

The surface morphology and the elemental composition of the NPMBs were analyzed before and after coating using a scanning electron microscope (SEM) coupled with energy dispersive X-rays (EDX) (JXA-840A, Electron probe micro-analyzer, JEOL, Tokyo, Japan) at 15 kV. In addition, the membranes were examined after their immersion in BSA to detect its accumulation on the surface of the membranes. The membranes were attached to the stainless steel sample holders with a carbon tape. The surfaces of the samples were then coated with a thin layer of gold using these holders, mounted on a piece of sputtering coating equipment. Afterwards, they were inspected using the scanning electron microscope and their microstructure features were captured.

#### 2.2.2. Transmission Electron Microscopy

High-Resolution Transmission Electron Microscopy (HR-TEM) (JEOL, Tokyo, Japan, JEM 2100, Electron Microscope, and HR-TEM) was used to examine the morphology and the size of the synthesized ZIF-8@GO composites in relation to GO. We ultrasonically mixed the powder samples with 10 mL of distilled water to produce a diluted suspension, and then we poured small droplets of this mixture into copper grids, which were subsequently allowed to dry in air on the top of the filter paper. The grids were put into the grid box, where the TEM machine was used to analyze them.

#### 2.2.3. Fourier Transform Infrared Spectroscopy (FTIR)

The functional groups of MOF nanoparticles with respect to GO were determined using the Fourier transform infrared spectrophotometer (model FT/IR-6100 type A, Jasco, International, Tokyo, Japan) at room temperature (25 °C) with a resolution of 4 cm^−1^. The samples were mixed with KBr powder (2 mg sample: 198 mg KBr). By applying a load on an evacuated mold for 5 min, the powder mixture was transformed into uniform discs, which were then examined using an FTIR instrument. The chemical alterations on the surfaces of coated polymeric membranes and alumina NPMBs were studied using FTIR. The Fourier transform infrared spectrophotometer (model FT-IR/ATR, Nicolet 6700 Thermo-fisher, Norristown, PA, USA)-based ATR method was used to record FTIR spectra. The measurements were made between 400 and 4000 cm^−1^.

#### 2.2.4. X-ray Diffraction Analysis (XRD)

The developed MOF nanoparticles were compared to GO using an X-ray diffraction analysis, which was carried out using a Bruker AXS, D8 Advance, Bruker, Leipzig, Germany X-ray diffractometer model with a Ni-filter and Cu radiation target, a tube voltage of 40 KV and a current of 40 mA. Using an agate mortar, 10 mg of a sample powder was thoroughly homogenized in cyclohexane. The XRD Perspex holder was then covered with this mixture, and it was dried at 70 °C for 5 min. The holder was then examined using an XRD with an examination speed of 2°/min and a 2Ɵ range of 2°–80°.

### 2.3. Contact Angle Measurement

One of the key characteristics that affect the ability of a membrane to resist fouling is its surface wettability. The hydrophilicity level of the membrane’s surface, in particular, reveals how various foulants behave when in contact with the membranes after fabrication. The angle formed between the tested membrane surface and the water droplet interface when the droplet is deposited vertically on the membrane’s surface is known as the contact angle (Ɵ). Using the OCA 15 EC optical contact angle device (Data Physics Instrument, Filderstadt, Germany), the contact angle of membranes coated with alumina NPMBs was determined in accordance with ASTM-D 7334-08 [30].

### 2.4. Atomic Force Microscopy (AFM)

The three-dimensional topography and roughness of the produced alumina NPMBs were investigated before and after coating by an atomic force microscope (AFM), (XE-100; Park Systems, Suwon, Republic of Korea). The atomic force probe’s scanning area was adjusted at 25 µm^2^ for this test, to collect the relevant parameters (Ra). The AFM was coupled with a WiTec alpha 300 R Raman Imagining Microscope. The AFM images were recorded in non-contractual mode using a silicon cantilever with 280–300 kHz and 42 N/m force constant.

### 2.5. In Vitro Studies

#### 2.5.1. Preparation of HEPES-Buffered ACSF

For a 10× stock solution 1.44 M NaCl, 100 mM HEPES buffer, 10 mM NaH_2_PO_4_ and 25 mM KCl were completely dissolved in deionized water (final volume 1000 mL). It was stored at 4 °C until it was used and it was discarded if precipitate appeared. For a 1× working solution, 10 mM glucose, 2 mM MgCl_2_ and 2.5 mM CaCl_2_ were completely dissolved in deionized water, then 1× of stock solution (100 mL) was added and the pH was adjusted to 7.3–7.4, and the final volume was completed to 1000 mL [31]. The pH of this ACSF was stable for about one month.

#### 2.5.2. Changes of ACSF Ions Concentrations

By soaking the NPMBs (0.7 × 0.7 cm) in ACSF, the ACSF ions concentrations were assessed before and after coating. The soaking conditions were a temperature of 37 °C kept for 28 days. Individual membranes were soaked in 50 mL of ACSF in the plastic containers. The withdrawal of 3 mL of the ACSF from each container at the same intervals was carried out to assess the concentrations of Al and Zn at certain time intervals (1, 3, 7, 14 and 28 days) by using an Agilent Technologies 5100 Inductively Coupled Plasma–Optical Emission Spectrometer (model, 720 ICP-OES, Agilent Technologies, Santa Clara, CA, USA) with Synchronous Vertical Dual View (SVDV), which was replaced with fresh ACSF. Spontaneously, the pH of the ACSF solution after each soaking time was recorded at set time intervals (1, 3, 7, 14 and 28 days).

### 2.6. Antifouling Tests

#### 2.6.1. Adsorption of Bovine Serum Albumin (BSA)

An organic fouling resistance test of all coated and uncoated NPMBs was carried out with a static adsorption technique, including bovine serum albumin (BSA) dissolved in an Artificial Cerebrospinal Fluid (ACSF) solution (1 g/L, pH 7.4). Static adsorption fouling was used to characterize the antifouling properties of the membranes [32]. The membranes were cut into regular shapes (0.7 cm × 0.7 cm), then coated with composite solution (PVDF/PVP with GO and GO-MOF) at two ratios (0.3 and 0.5 wt.% of GO) and were immersed in BSA solution (1 g/L, pH 7.4) at a temperature of 37 °C. At predetermined times (1 h, 3 h, 5 h, 1 d, 2 d, 5 d, 14 d) 3 mL of BSA solution was separated out and replaced with a fresh one. The concentrations of BSA solution were estimated before and after adsorption using a UV spectrophotometer (JascoV-730 Jasco International, Tokyo, Japan) at a wavelength of 260 nm by applying the equation of the predetermined standard curve for the BSA. Each sample was assessed in triplicate and their average values and the adsorption % were calculated from Equation (1).

(1) $$\%\;BSA\;adsorption=\left(\frac{Intial\;conc.\;of\;BSA-Final\;conc.\;of\;BSA\;in\;solution}{Intial\;conc.\;of\;total\;BSA}\right)\times 100$$

#### 2.6.2. Changes in pH Values

At predetermined intervals (1, 3, 7, 14 and 28 days), the change in pH of BSA was measured as a function of time. The pure and coated membranes were extracted from the BSA solution; furthermore, they were washed and dried for observation using SEM coupled with EDX for the assessment of the rejection and precipitation of BSA on the membranes’ surfaces as well as the relation between pH and BSA rejection.

### 2.7. Anti-Biofouling Activity against Bacteria

The antimicrobial and anti-biofouling activities of the membranes were assessed via light microscopy (LM) using the model bacteria Gram-negative *Escherichia coli* ATCC-25922 and Gram-positive *Staphylococcus aureus* ATCC-6538. The American Type Culture Collection (ATCC, Rockville, MD, USA) provided these microbes. By sub-culturing the bacterial strains in a new nutrient broth medium for 24 h prior to the test, the bacteria were revived for the bioassay. A bacterial suspension (1 mL) was added to fresh nutrient broth medium (9 mL), and the bacteria were allowed to grow for 4 h until they attained an optical density of 1.4 at 600 nm. The cells were centrifuged at 10,000 rpm for 10 min before being rinsed with phosphate-buffered saline solution and diluted to 107 CFU/mL. This test was carried out similarly to the assay described in previous research with various modifications [32,33,34].

Circular membrane pieces with an area of 0.88 cm^2^ (a diameter of 0.7 mm) were cut and immersed in bacterial suspension (10 mL) for 15 min. The bacterial suspensions were discarded after incubation, and the membranes were placed into new tubes containing 10 mL of phosphate-buffered saline. The antibacterial activity of the membranes was further investigated using the LM test, which used a live/dead Gram-staining technique to detect the viability of bacterial cells on the membranes’ surface [35]. In brief, membranes were incubated in *E. coli* or *S. aureus* suspensions for 15 min before the surface-attached bacteria were stained with Gram-staining for dead and live cells, respectively. The photos were then captured using a light microscope (C2, Nikon Corporation, Tokyo, Japan).

### 2.8. Drug Delivery through Different Membranes

#### 2.8.1. Drug Loading

For drug loading onto AAO membranes (NPMBs), uncoated NPMBs membranes with dimensions 1 cm × 1 cm were loaded with 500 µg donepezil solubilized in 50 µL of a CSF per membrane and left to air dry, then coated with a spray dryer with different polymeric solutions against uncoated NPMBs [36].

#### 2.8.2. Drug Release

In vitro donepezil release was assessed in CSF under dynamic (shaking) conditions. Samples were soaked in 50 mL of CSF, and after that 3 mL of the solution was withdrawn at specific time points, namely, 2, 3, 6, 8, 24 h, 2 and 7 days. Every time, the solution was replaced with fresh CSF in order to maintain the same conditions throughout the whole experiment [36]. The collected solutions were frozen at −20 °C. Using a UV spectrophotometer with a 230 nm wavelength, the amount of donepezil released into the solution was quantified. The drug concentration was also calculated using the previously established standard curve. The drug performance profiles were matched against different mathematical models—the zero-order model [37], Higuchi’s model [38] and Koresmyer–Peppas model [39]—using the following equations.

The zero-order model, Equation (2):

Q_t_ = Q_0_ + K_0_ t (2)

Higuchi’s model, Equation (3):

Q_t_ = k_Ht_^1/2^ (3)

Korsmeyer–Peppas model, Equation (4):

Q_t_/Q_0_ = K_k_t^n^ (4)

where Q_t_ is the cumulative drug released in time t; t represents the leaching time in hours; Q_0_ is the original amount of the drug in the sample; K_0_, K_H_ and K_K_ are the zero-order, Higuchi and Korsmeyer–Peppas dissolution rate constants, and n is the kinetic exponent.

## 3. Results and Discussion

### 3.1. Scanning Electron Microscopy

**Figure 2:**
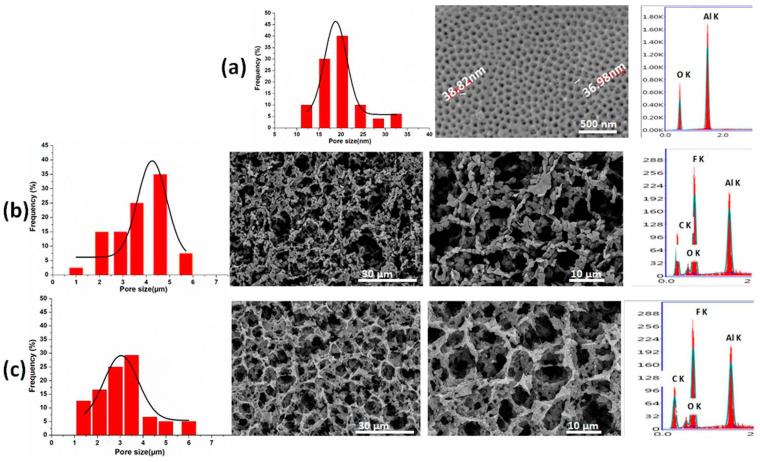
SEM/EDX analysis of (**a**) alumina NPMBs, (**b**) alumina NPMBs coated with PVDF/PVP loaded with GO and (**c**) alumina NPMBs coated with PVDF/PVP impregnated with ZIF-8 MOF@GO, and their corresponding histograms depict the pore sizes, constructed by image j (1.54 g).

Figure 2 illustrates the obtained nanoporous membrane of alumina as well as the micro-porous 3D structures of the fabricated coating. The fabricated NPMBs (Figure 2a) demonstrated a homogenous nanoporous structure with a pore size in the range of 36 to 39 nm. Its corresponding EDX chart only showed the presence of Al and O elements, confirming the purity of the alumina NPMBs achieved. The formed layers were found to have a relatively homogeneous distribution of micropores, which was well-arranged, and the coating layer showed the presence of ZIF-8 MOF@GO in the PVDF/PVP coating (Figure 2b,c). The inclusion of PVP increases the membrane’s roughness by acting as a pore-forming reagent to accelerate the rate of phase inversion during the formation of the membrane and lower PVDF’s hydrophobicity. The incorporation of GO in PVDF/PVP membranes influences the pore structure of PVDF, which is related to the formation of hydrogen bonds between GO and PVP [40,41]. The uniform and dense pore structure distributed on the membrane’s surface was greatly enhanced due to the addition of GO/Zn MOF nanoparticles to the polymeric membranes. Eventually, thin, porous and hydrophilic surface coatings were constructed on the NPMBs to decrease their fouling [40]. The presence of the Zn element was not proven with EDX analysis (Figure 2c) because of its complete impregnation within the PVDF/PVP matrix and due to its low concentration. However, its effect was noted on the morphology of the pore walls of the fabricated coating layer, as they became more rough compared with the PVDF/PVP matrix loaded with GO alone. The images provided, with their corresponding histograms, depict the pore sizes constructed by image j (1.54 g)., showing the average pore size distribution, which reached maximums of 40, 35 and 30 for alumina NPMBs, PVDF/PVP loaded with GO and alumina NPMBs coated with PVDF/PVP impregnated with ZIF-8 MOF@GO, respectively. These findings demonstrate that the surface modification method we proposed could simultaneously increase the membrane flux and antifouling abilities when implanted in cerebrospinal fluid, potentially providing a new insight into the fabrication of high-performance membranes used for active molecule delivery or the removal of unwanted molecules (amyloid beta plaques in Alzheimer cases) without any foulant accumulation.

### 3.2. Characterizations of the Coating Materials

#### 3.2.1. Transmittance Electron Microscopy (TEM)

**Figure 3:**
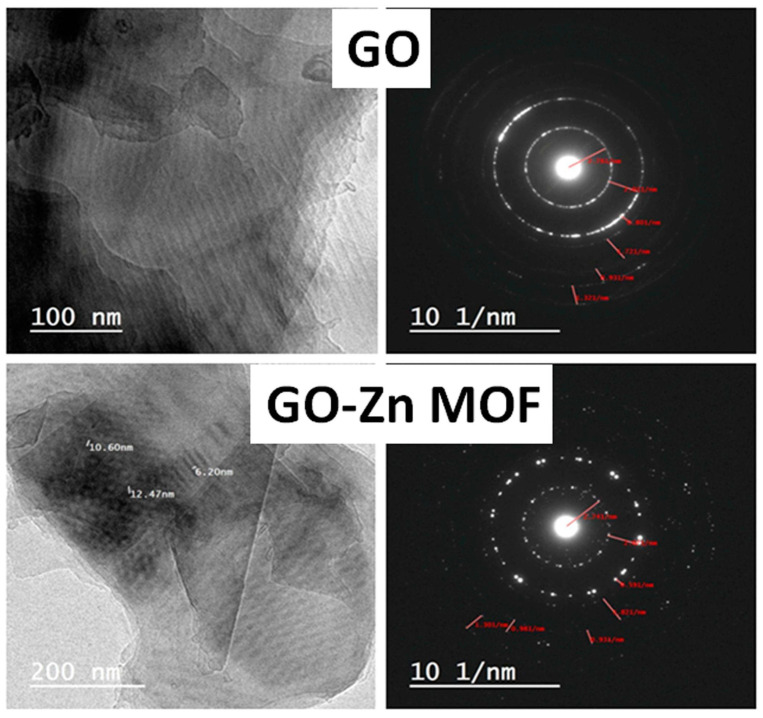
TEM images recorded for GO and GO-Zn MOF along with their corresponding diffraction patterns.

The textures of the nanosheets were observed by TEM, and are shown in Figure 3. It was shown that the ZIF-8/GO nanocomposites have a unique structure. The GO alone exhibits a characteristic, translucent and distorted laminar structure, while hexagonal ZIF-8 crystals with a few irregular squares/rectangles are consistently stabilized on the GO surfaces in the case of GO-Zn MOF nanosheets. Furthermore, more ZIF-8 crystals were produced on the borders of the GO nanosheets than in the center. The GO sheets appeared to facilitate nucleation as a growth matrix for ZIF-8 crystals. The hydrogen bonding interaction between the 2-methylimidazole in ZIF-8 and the abundant hydroxyl, carboxyl, and epoxy groups of GO is due to the deposition of ZIF-8 onto the GO nanosheets. As a result, the use of the in situ growth strategy in decoration is recommended, since ZIF-8 was homogeneously distributed onto the surface of GO, which may be the ideal structure for ZIF-8/GO hybrid nanosheets to exhibit their maximum activity. Under the decoration method of ZIF-8, nanocrystals are dispersed with a very small particle size of 5–20 nm. Similar results have been reported for ZIF-8 with enhanced antimicrobial activities endowed by graphene oxide [42]. Moreover, the diffraction patterns of GO samples before and after decoration are represented in Figure 3. These patterns have confirmed the presence of ZIF-8 nanoparticles on the GO nanosheets, as can be inferred from the illuminated spots located in both samples. However, these illuminated spots were found to be distributed more homogeneously in the diffraction pattern of GO-Zn MOF, which agrees with the SEM results for the coated layers.

#### 3.2.2. Fourier Transform Infrared Spectroscopy (FTIR) of GO and GO-Zn MOF Composites

**Figure 4:**
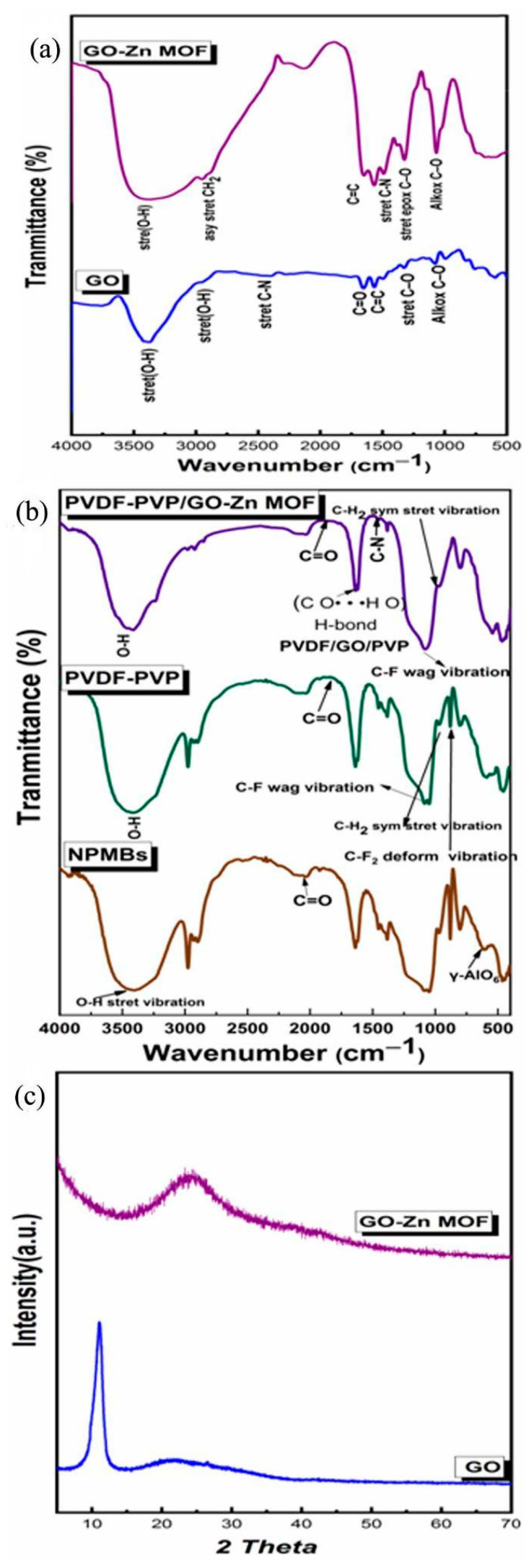
(**a**) FTIR spectra of the fabricated GO-Zn MOF with reference to GO, (**b**) FTIR spectra of fabricated coatings compared to pure NPMBs. (**c**) XRD curves recorded for the fabricated GO-Zn MOF with reference to GO.

The FTIR spectra of GO and GO-Zn MOF samples are shown in Figure 4a. Oxygenated functional groups appear as broad bands around 3416.35 and 3390.53 cm^−1^ for both GO and GO-Zn MOF samples, respectively. These bands are assigned to the O–H stretching vibrations of water and hydroxyl groups in graphene oxide. The O–H stretching vibrations in ZIF-8/GO nanosheets are responsible for making them significantly weaker in GO-Zn MOF compared to native GO, and this proves the presence of ZIF-8/GO nanocomposites in the polyamide thin layer. The GO sample also showed an absorption band of the carboxyl C=O (1756.98 cm^−1^), along with apparent C=C bands of aromatic rings within the GO carbon skeleton structure (1653.12 and 1648.94 cm^−1^) for GO and GO-Zn MOF, respectively. An epoxy C–O stretching vibration (1326.37 and 1324.28 cm^−1^) for GO and GO-Zn MOF is also observed. Alkoxy group C–O (1077.37 and 1067.22 cm^−1^) is seen for GO and GO-Zn MOF, respectively, which confirms the presence of oxygen-containing functional groups, such as C=O and C–O; furthermore, it confirms that the graphite was in an oxidized form [25]. As shown in Figure 4a, GO-Zn MOF exhibits a new band at 1381.95 cm^−1^. This band corresponds to the amide structure of the polyamide layer that is generated during interfacial polymerization, and belongs to the C–N stretching vibrations. This suggests that the polyamide layer is formed between GO and ZIF-8 in nanocomposites. The IR bands allocated at 2959.10 and 2952.01 cm^−1^ are assigned to the asymmetric CH_2_ stretching of GO and GO-Zn MOF, respectively. Also, this band is a characteristic band of tetrahedral carbon–hydrogen bonds that maybe adsorbed from the surroundings.

#### 3.2.3. FTIR Analysis of NBMBs and Coated Membranes

The FTIR spectra of the NPMBs and PVDF/PVP and ZIF-8 MOF @GO fabricated coatings are depicted in Figure 4b, to ensure the presence of different functional groups representative of the functional groups for each coating. The Al_2_O_3_ NPMBs showed bands at 607.467 cm^–1^, which are assigned to γ-AlO_6_. The broad band at 3428 cm^–1^ and the weak band at 1639 cm^–1^ are due to the water adsorbed from the surroundings. This suggests the relative stability of the prepared NPMBs, as was earlier reported [43]. The PVDF/PVP coatings showed a band located at 2975.62 cm^−1^ corresponding to the CH_2_ symmetric stretching vibration of PVDF. The peak at 1400.07 cm^−1^ represents CH_2_ molecular vibration. The band observed at 1083.8 cm^−1^ is related to C–F molecular vibration and that at 879.381 cm^−1^ is related to CF_2_ deformation vibration (879.38165 and 802.24 cm^−1^). The C=O group from PVP was noticed at wave numbers 1816 and 1770 cm^−1^.

The bands for the ZIF-8 MOF@GO functionalized coatings suggest CH_2_ vibration, at 970 and 798 cm^−1^, along with a band located at 1384.6 cm^−1^ that belongs to O–H deformation. An obvious band was noticed at 1772 cm^−1^ corresponding to the C=O group, proving the presence of GO and PVP in the blended membrane (PVDF/GO/PVP) due to the formed hydrogen bonds (C O· · ·H O). The intensity of the C–N band at 1454 cm^−1^ in EDC samples indicates that the EDC-crosslinked samples contained certain amounts of amide bonds due to the EDC crosslinking. The presence of N-Hydroxysuccinimide (NHS) manifested remarkable absorption peaks at 1637 cm^−1^ due to the N–O bond [32]. As a result, the inclusion of GO and/or PVP in the PVDF polymer matrix affected the crystalline phase behavior dramatically. As a result, after being blended with GO and PVP, the PVDF-based coatings in this study were projected to become more hydrophilic [34,35].

#### 3.2.4. X-ray Diffraction (XRD)

Figure 4c shows XRD patterns of GO and GO-Zn MOF hybrid nanosheets. GO exhibited its most intense peak at 2Ɵ = 11.17640°, corresponding to the (002) plane and representing an interlayer distance of 7.916 [25]. The composite material GO-Zn MOF was characterized by an amorphous phase of the ZIF-8/GO nanocomposite, which reveals that there is a strong interaction between the ZIF-8 and the GO, causing ZIF-8@GO to exhibit an amorphous nature.

### 3.3. Contact Angle

**Figure 5:**
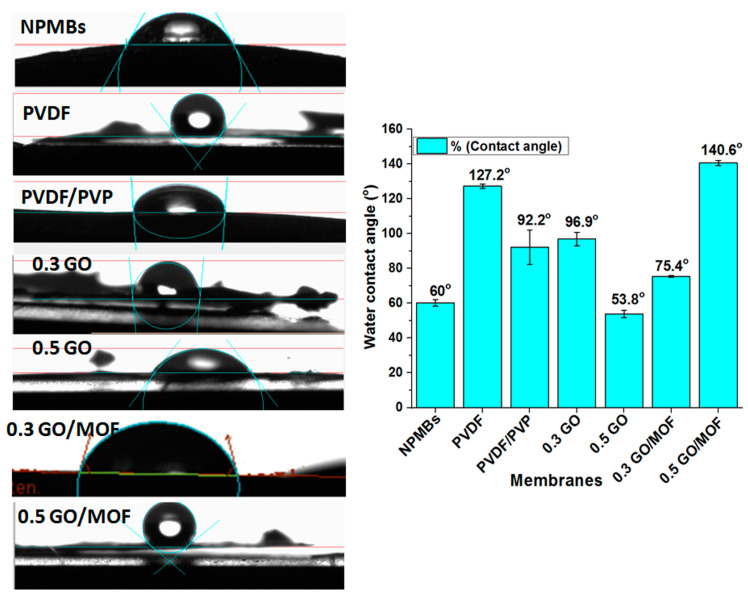
Contact angle images and values of alumina NPMBs and coated membrane surfaces.

The contact angle measurements were used to determine the surface hydrophilicity of the coated membranes. The contact angle (CA) between water and the membrane surface was measured using a contact angle measurement instrument. A water droplet was deposited on the membrane surface and monitored until it stopped changing, and the contact angle value was recorded as the short measurement period. For each sample, the average value was calculated after at least three tests with a deviation of less than 5%. According to Figure 5, the recorded contact angles of various membranes revealed a good antifouling behavior for GO-Zn MOF/PVDF/PVP functionalized coatings because of the presence of GO with abundant oxygen-containing groups and water-soluble PVP acting as a pore-forming reagent. The synergetic effects between GO and PVP can improve the antifouling properties of functionalized coatings. Furthermore, the compact water layer (hydration layer on the functionalized coatings) hampered the proteins’ or biofoulants’ deposition on the membranes. This, in turn, would improve the antifouling performance [35,36].

The variation in the contact angle is suggested to be due to the different compositions and concentrations. The PVDF membrane showed the highest contact angle value among non-functionalized membranes because of its hydrophobic nature [41]. The presence of a hydrophilic material such as GO and MOF causes a decrease in the contact angle [42,43]. The lowest contact angle was 0.5% for GO (53.8°), which is explained by the increase in GO concentration, causing an increase in the hydrophilicity that resulted in a decrease in the contact angle [44,45,46]. Thus, the incorporation of GO and MOF promotes hydrophilicity from 127° to 53.8°. However, it is noticed that there was an increase of 0.5% in MOF (140.6°), and this can be explained as resulting from the agglomeration of the nanoparticles in the polymer matrix, thus blocking their sites of action, and there were some factors that could reduce the positive effects of the incorporation of GO and MOF, such as the porosity.

### 3.4. Atomic Force Microscopy

**Figure 6:**
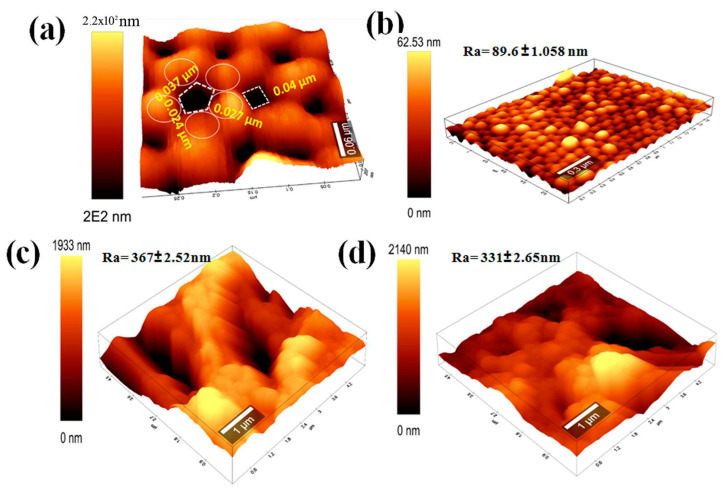
Surface AFM images of (**a**) non-coated AAO NPMBs, (**b**) PVDF/PVP, (**c**) 0.5 PVDF/PVP GO and (**d**) 0.5 PVDF/PVP GO-MOF, n = 3.

Figure 6 depicts the three-dimensional topography and correlating surface roughness of the membrane samples. Figure 6a illustrates the topological surface profiles of AAO NPMBs. A highly ordered AAO structure emerged, composed of nanopores with a two-dimensional tetragonal, circular and hexagonal closed packed pattern, surrounded by four or six bumps. This suggests the possibility of the formation of multiple shapes of pores. The pore depth was hardly measured due to the limited penetration of the AFM tip into the pore. The addition of nanoparticles to the membrane matrix frequently has a considerable effect on the surface roughness [47]. The average roughness (Ra) values of PVDF/PVP, 0.5 PVDF/PVPGO, and 0.5 PVDF/PVPGO-MOF membranes were 89.6 ± 1.058, 367± 2.52, and 331± 2.65 nm, respectively. Because of the craggy valleys generated by the introduction of zinc MOF on the membranes’ surfaces, the average roughness was gradually improved by increasing the MOF-GO content, which is compatible with the SEM results and prior reports in the literature [47]. Furthermore, in the case of general hydrophilic membranes, a larger surface roughness promoted the permeability due to an increased effective filtration area.

Figure 6 displays the mean surface roughness (Ra) and surface AFM images of the PVDF/PVP, 0.5 PVDF/PVP GO, and 0.5 PVDF/PVP GO-MOF membranes. As shown in Figure 6, the tidy PVDF/PVP membrane (M1) exhibits an Ra of approximately 89.6 ± 1.058 nm, and the addition of PVP into the PVDF matrix effectively raises the surface roughness of the membrane over that reported in the previous work [41], which was 64.3 nm. This membrane has greater hydrophobicity characteristics than those coated with Zn MOF. The membranes with the highest surface roughness were those made of 0.5 PVDF/PVP GO and 0.5 PVDF/PVP GO-MOF. This is because GO, GO-MOF, and PVP have synergistic effects in terms of increasing hydrophilicity, which raises the pace at which non-solvents and solvents are exchanged during the inversion process, and results in a nanocomposite membrane with a higher degree of roughness. Accordingly, a high surface roughness in the case of hydrophobic membranes results in poor antifouling properties, but high surface roughness in the case of hydrophilic membranes produces cavities, and thereby increases the membrane surface area, which enhances antifouling properties [47,48]. The relatively higher roughness can improve the efficient filtration area, which increases the antifouling property.

### 3.5. Stability of Composite under ACSF Conditions

#### 3.5.1. ACSF Ions Concentrations

**Figure 7:**
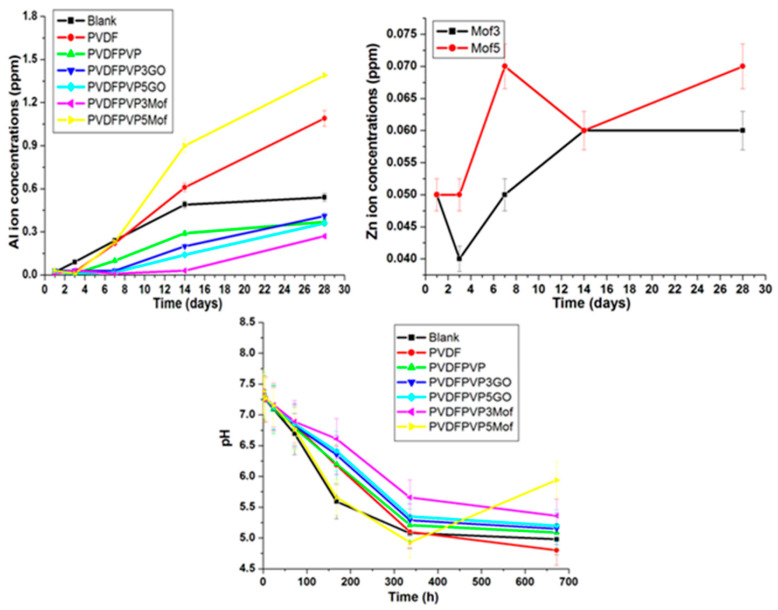
The Al and Zn ions released, as well as pH, after the soaking of coated and non-coated NPMBs in ACSF for 28 days.

The release of Al ions increases over 28 days up to 1.4 ppm (1.4 µg/mL). The Department of Agriculture (USDA) Nationwide Food Consumption Survey has estimated daily aluminum intakes of 0.10–0.12 mg Al/kg/day for adult (25–30 and 70+ years old) males and females. This means that the detected release represents very minor traces of Al that do not affect human health [49]. The best coating materials that do not allow any Al ions to be released are PVDF/PVP, PVDF/PVP 0.3 GO, PVDF/PVP 0.5 GO and PVDF/PVP 0.3 GO Zn MOF (see Figure 7). Moreover, the release of Zn ions increases to 0.06 and 0.07 ppm after 28 days. The two compositions that contain the Zn element (MOF) show almost the same final release rates. The rate of release of Zn ions in the case of 5% MOF is slightly more than 3% MOF because of the higher percentage of Zn existing in 5% MOF compared to 3% MOF. Also, the released Zn ions were found in traces that do not affect human health (see Figure 7). Furthermore, the pH values decreased gradually up to 5–5.5. The decrease in pH was found to be correlated with a small dissolution of Al and Zn ions, which resulted in increased acidity (see Figure 7).

#### 3.5.2. Antifouling Tests

##### Adsorption of Bovine Serum Albumin

**Figure 8:**
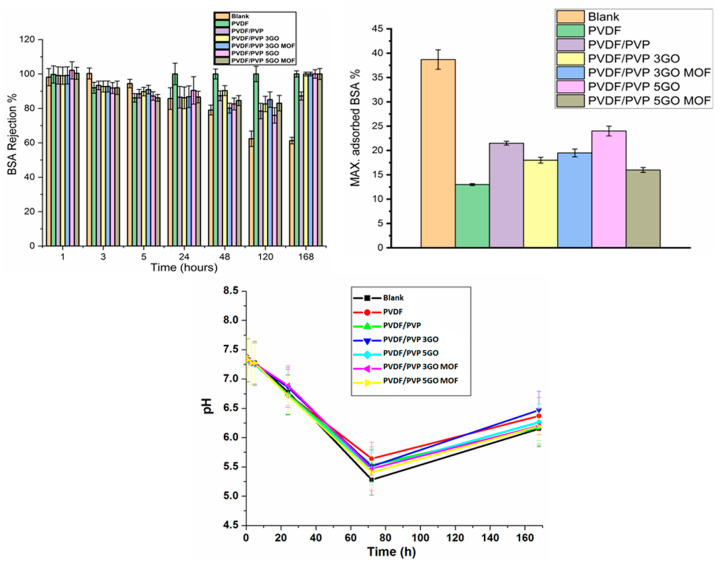
BSA rejection (%) at time intervals (1 h, 3 h, 5 h, 1 d, 2 d, 3 d, 7 d), the maximum adsorbed BSA (%), and the pH of BSA during the whole incubation time (7 days).

On the first day, coated and non-coated AAO NPMBs demonstrated 100% rejection; however, these rejection percentages remained the same for the coated membranes, especially those coated with PVDF/PVP loaded with 0.5 wt.% GO, 0.5 GO Zn MOF, 0.3 wt.% GO and 0.3 GO Zn MOF till the last day of incubation (day 7). On the other hand, the non-coated AAO NPMBs showed a marked decline in the rejection percentage due to the biofoulant accumulation, which started from the 5th hour and continued through all incubation time intervals (see Figure 8). The value of pH decreased gradually over 3 days up to 5.5, then increased to around 6.5 at 7 days. The rejection of BSA was accompanied by a low pH, of around 7 [50,51]. Thus, the pH of the BSA solution confirms the BSA’s rejection (see Figure 8).

The maximum adsorbed BSA (%) during the whole incubation time (7 days) on all tested membranes shows that PVDF comprised a super hydrophobic surface, while it showed the lowest amount of adsorbed BSA (13%), and PVDF/PVP 0.5 GO Zn MOF showed the next lowest amount of adsorbed BSA (16%). Moreover, the PVDF/PVP and PVDF/PVP 0.5 GO membranes showed the highest rates of BSA adsorption percentage (21% and 24%, respectively), as compared to all polymer-coated membranes, while the differences in BSA adsorbed on PVDF/PVP 0.3 GO and PVDF/PVP 0.3 GO Zn MOF were non-significant (18% and 19.5%, respectively). Finally, the AAO non-coated nanoporous membrane (blank) showed a marked biofoulant accumulation on its surface, with a maximum adsorbed BSA (%) of 39% of the concentration of BSA that was utilized (1 g/L ACSF) [22].

##### SEM of the Membranes after BSA Static Immersion

**Figure 9:**
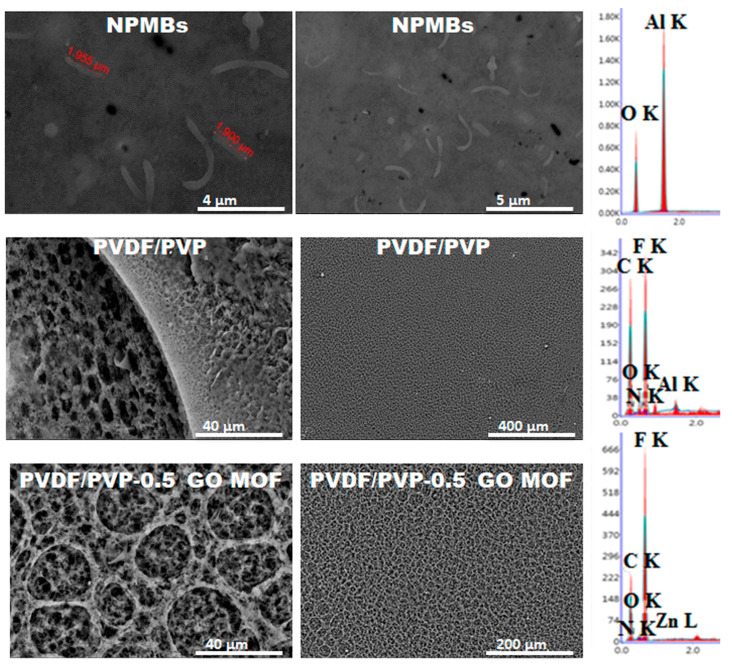
SEM images and their corresponding EDX after immersion for 7 days in static BSA solution.

Figure 9 shows nanoporous membranes after their immersion for 7 days in static BSA solution dissolved in ACSF, to mimic the natural cerebrospinal fluid treatment conditions. The uncoated Al_2_O_3_ nanoporous membrane showed a marked accumulation of BSA and biofoulant on it is surface, while PVDF/PVP showed lower amounts of BSA adsorbed on the polymer-coated membrane’s surface. Also, the SEM images show a lateral view of the membrane, revealing a good porous structure. The PVDF/PVP 0.5 GO Zn MOF membrane was nearly cleared of BSA via adsorption, and it showed a well-organized texture upon immersion for 7 days in a BSA solution.

These findings demonstrate that the surface modification method we proposed using GO-MOF can simultaneously increase the membrane rejection and antifouling ability during implantation in cerebrospinal fluid, providing new insights into the fabrication of high-performance membranes for the removal of proteins and biofoulants in drug delivery applications. EDX elemental analysis was used to determine the membrane’s elemental composition. The results in Figure 4b for non-coated NPMBs show main peaks corresponding to Al and O, and for PVDF/PVP, the main peaks correspond to N, C, Al, F and O, confirming the presence of both polymers. In the case of 0.5 PVDF/PVP GO-MOF, the presence of main peaks corresponding to N, C, F, Zn and O within the matrix confirms the presence of zinc MOF within the membrane’s coating.

##### Anti-Biofouling Activity against Bacteria

**Figure 10:**
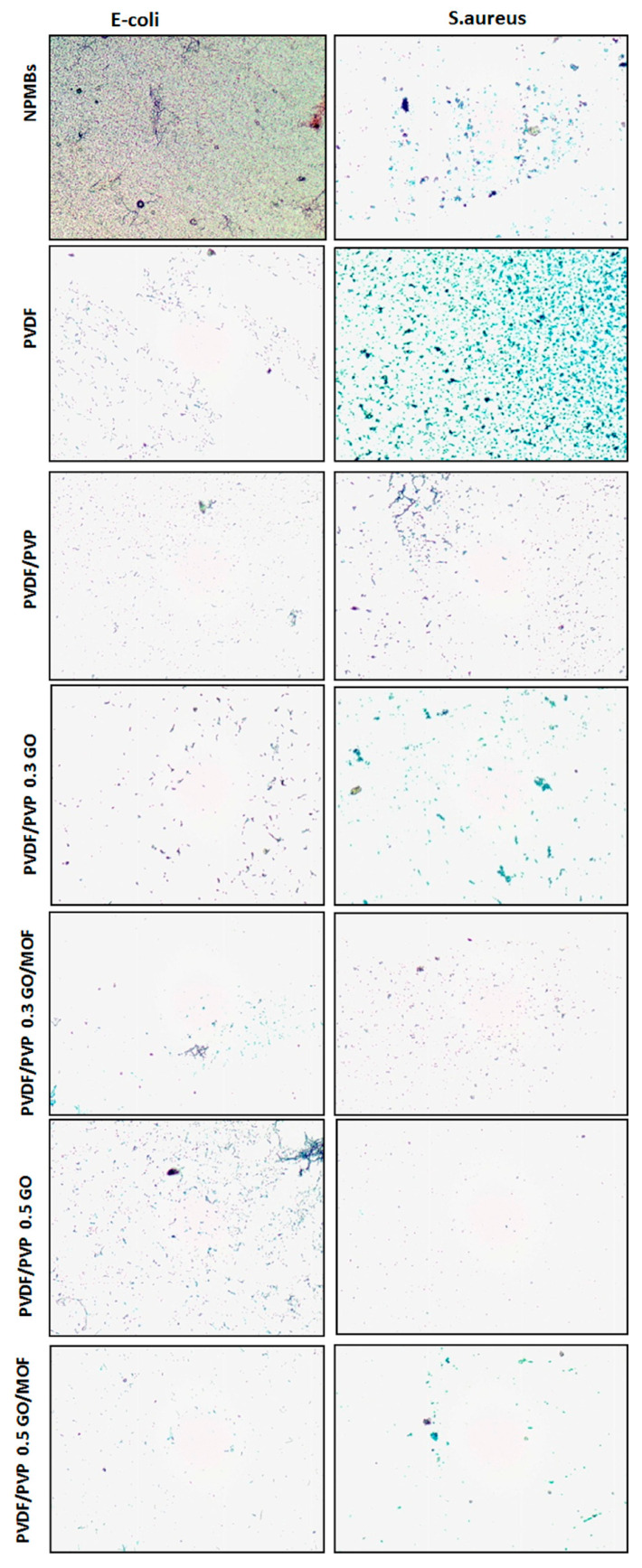
The alumina NPMBs before and after different treatments of the coatings exposed to *Staphylococcus aureus* and *Escherichia coli*.

Figure 10 shows photos of the alumina NPMBs before and after different treatments of the coating exposed to *Staphylococcus aureus* ATCC-6538 and *Escherichia coli* ATCC-25922 bacterial suspensions. Static contact experiments using the LM test were undertaken to evaluate the membranes’ anti-biofouling characteristics. In these tests, *E. coli* and *S. aureus* were utilized as model Gram-negative and Gram-positive bacteria, respectively. The LM results reveal that the majority of the bacteria in contact with the NPMBs and PVDF-coated membranes survived and accumulated on the surfaces, while a majority of the bacterial cells in contact with PVDF/PVP, GO and GO/MOF membranes were inactivated and detached from the membranes’ surfaces, with the most notable ratio of 0.5 wt.% seen for carbonaceous material. As previously reported [37], a synergistic action involving graphene oxide and ZIF-8 is one of the potential mechanisms playing an essential role in the antibacterial and anti-biofouling activities of GO/MOF membranes. It was concluded that the GO/MOF-coated membranes have a stronger antimicrobial activity and a more acute anti-biofouling property, which agrees with the BSA antifouling results.

Theoretically, bacterial inactivation by GO involves a direct puncturing of the cell membrane, oxidative stress, phospholipid extraction from the lipid bilayer, and the attachment of graphene sheets to the cell surface. Furthermore, the imidazole ring in ZIF-8 has an antibacterial activity, and imidazole derivatives have a wide range of biological functions [50]. Imidazole’s antibacterial effects stem from the rupture of liposomes, which are made up of phospholipids carrying unsaturated fatty acids. The production of zinc ions increases ZIF-8’s antibacterial action due to metal ions’ intrinsic antimicrobial properties. MOFs can operate as a reservoir for metal ions, resulting in a prolonged antibacterial activity similar to that reported for metal/metal oxide nanoparticles (NPs) [40,52,53].

### 3.6. Drug Delivery

**Figure 11:**
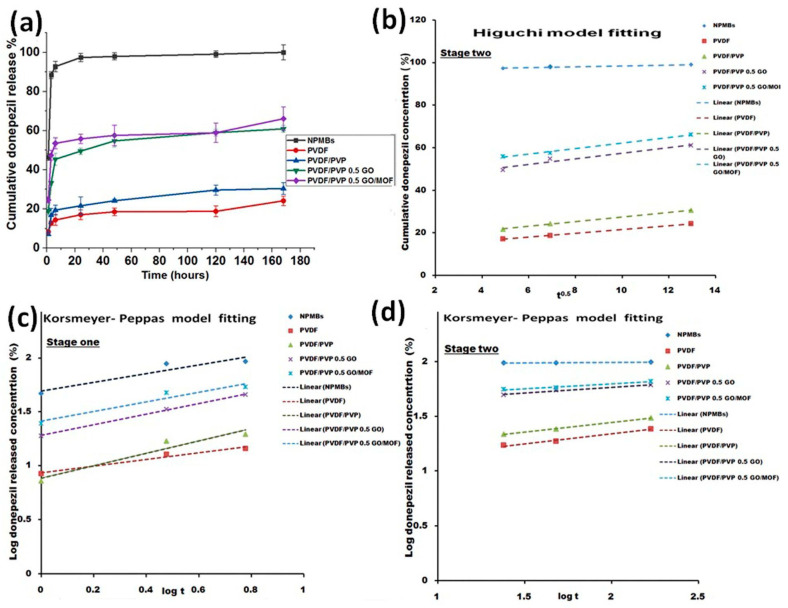
(**a**) In vitro release of donepezil from different membranes (mean ± standard deviation, n = 3). (**b**) The application of the Higuchi model to the profile of drug release from all membranes from 6 h until the end of the release period (7 days). (**c**,**d**) The application of the Korsmeyer–Peppas model to the profile of drug release from all membranes throughout both stages.

The cumulative release profiles of donepezil from uncoated and polymer-coated AAO NPMBs are shown in Figure 11a. The release profiles of the drug from the samples have two stages—a fast one lasting for the first 6 h (the fastest rate of release being from uncoated membranes as compared to polymer-coated ones), followed by a stage of sustained release via pores. Thus, the sustained release of the drug was achieved by all NPMBs. However, the GO-modified membranes were the most capable of delivering the drug in a controlled manner (over 7 days).

For uncoated nanoporous membranes, the early burst of release over the first 6 h (stage one) released about 92.7 ± 2.6% of the total amount of loaded drug. This means that most of the loaded drug was leached from the membrane surface in just 6 h, which could cause a toxic dose to the patient, and this could be considered as a drawback limiting the utility of uncoated NPMBs as a drug delivery system for donepezil. The final cumulative percentage of drug release during both stages was about 99 ± 3.7% of the loaded drug, i.e., the uncoated NPMBs released the entire loaded drug in less than 7 days.

On the other hand, polymer-coated PVDF and PVDF/PVP membranes released about 14.4 ± 2.7% and 19.4 ± 2.4% of the drug, respectively, in the first 6 h, while the final cumulative percentages of drug released during the whole release period (7 days) were about 24.2 ± 2.3% and 30.4 ± 3.0%, respectively. Finally, the rates of drug release from the membranes modified with 0.5 GO and 0.5 GO/MOF were about 45.4 ± 2.94% and 53.5 ± 2.9%, respectively, in the first 6 h, which percentages could represent an effective therapeutic dose that will inhibit acetylcholinesterase activity [27]. The final cumulative percentages of drug released during the whole release period (7 days) were about 60.95 ± 3.7% and 66.12 ± 5.9%, respectively. Comparing the early stage of release from the polymer-coated and modified membranes with that from uncoated NPMBs gives a clear indication that polymer-coated membranes could deliver a moderate controlled drug dose. This system would provide safer and less toxic doses for patients, enable self-control, and regulate the drug release profile. Accordingly, regarding the polymer-coated membranes, PVDF and PVDF/PVP were still loaded with 75.8% and 70.6%, respectively, and regarding the GO-modified samples, PVDF/PVP 0.5 GO and PVDF/PVP 0.5 GO/MOF were still loaded with about 39% and 36% of the drug, respectively. Examining the profiles of the release of the drug from those samples, we see that both samples were able to deliver the drug for longer than 7 days compared with the NPMBs. Thus, polymeric coated NPMBs could be used as a controlled localized donepezil delivery system for Alzheimer treatment.

#### Drug Releasing Kinetics

We applied different drug models, i.e., the zero-order model [37], Higuchi’s model [38], and the Korsmeyer–Peppas [38] model, to the two stages of drug release, and the release constants and correlation coefficients of these models are illustrated in Table 1 and Table 2 and Figure 11. The Higuchi drug release model was the most fitted to stage two of drug release, lasting from 6 h until the end of the release period (7 days), as shown in Figure 11b–d. Linear regression analysis was performed by fitting a straight line through the release data, and high correlation coefficients (R^2^ > 0.90 that ranged from 0.993 to 0.998) were detected following the regression analysis of the drug-release data, indicating that the drug was released from all samples via a diffusion-controlled mechanism.

The rates of drug release from other membranes through stage 1 and stage 2 were estimated using the Korsmeyer–Peppas model, which was highly fitted (indicated by higher R^2^ values, R^2^ > 0.9). In the analysis of stage one of drug release, lasting up until 6 h, the drug was released via quasi-Fickian diffusion (n < 0.45) (i.e., semi-controlled release) in the case of NPMBs and PVDF-coated membranes, while it was released via anomalous (non-Fickian) diffusion—indicating drug release through the swelling and erosion of polymeric membranes—in the case of PVDF/PVP, PVDF/PVP 0.5 GO and PVDF/PVP 0.5 GO/MOF membranes (0.45 < n < 0.89). In stage two of the drug release analysis, all n values for all membranes were n < 0.45, indicating quasi-Fickian diffusion (n < 0.45).

Some successful previous examples of modified nanoporous membranes being used in biomedical applications, including cardiology and tissue engineering, have been studied using different drugs and modification techniques [28]. In this study, the evaluation of donepezil loading and release from GO and GO-Zn MOF-modified polymeric-coated alumina NPMBs was studied for the first time. The findings demonstrate that the coating of the alumina NPMBs’ surfaces with PVDF and PVDF/PVP reduced the burst release behavior and prolonged the profile of sustained release from the developed drug delivery system. On the other hand, modifying the polymeric coatings with GO and GO-Zn MOF enabled us to control both the burst and sustained drug release concentrations to levels that could offer an effective therapeutic dose as part of an appropriate drug delivery system.

## 4. Conclusions

Alumina NPMBs were successfully fabricated using a two-step electrochemical anodization technique with a homogeneous porous structure possessing a pore size of 35 ± 5 nm. A PVDF/PVP composite polymer matrix loaded with carbonaceous fillers was successfully incorporated with Al_2_O_3_ NPMBs, along with functionalization using a spin-coater. The ATR-FTIR spectra of coated membranes show characteristic bands of polymers and carbonaceous fillers, as well as their possible linkages. This, in turn, proved that the method used for coating purposes has no effect on the chemical integrity of the coating components. The NPMBs demonstrated a degree of hydrophilicity in the functionalized membranes that was controlled by the formation of a hydration layer on the functionalized membrane’s upper surface, as confirmed by the results regarding the contact angle, which is expected to act as a barrier to reduce protein or biofoulant deposition on the membrane’s surface. SEM analysis with EDX ensured the formation of microporous membranes with ordered pores in a 3D topography, which will be favorable for protein antifouling and the circulation of biological fluids (ACSF in this case). The in vitro release of Al and Zn ions in ACSF was also determined, and they were found below the acceptable limits in the human body. Moreover, changes in pH of the ACSF with time were monitored throughout the immersion period, and the presence of coating materials had a minimal effect in terms of reducing pH. The antifouling effect was found to be dependent on the compositions of the coating materials regardless of the roughness degree, as confirmed by AFM. The enhancement of the antifouling property of the AAO NPMBs was confirmed by SEM coupled with EDX and BSA (%) rejection. The anti-biofouling properties of the membranes were also evaluated via static bacterial contact experiments through the LM test. Synergistic effects involving GO and Zn MOF were identified as a possible pathway playing an important role in the antimicrobial and anti-biofouling activity of GO/MOF. Finally, it was concluded that the GO/MOF-coated membranes had a stronger antimicrobial activity; their acute anti-biofouling property matched with BSA’s antifouling results, and would enable donepezil HCl to be delivered in a controlled manner over a time period longer than that for the uncoated NPMBs. The most promising antifouling drug delivery membranes based on these structuring techniques were made of PVDF/PVP loaded with both GO and MOF-derived ZnO.

## Figures and Tables

**Table 1:** Release kinetics parameters of donepezil loaded onto uncoated and polymer-coated alumina NBMBs. Stage 1.

**Formula Code**	**R^2^-Value ^†^**			**Korsmeyer–Peppas Model**		**n**	**RE_0–6 h_^‡^** **(%)**
	Zero-Order	Fickian Diffusion	Korsmeyer–Peppas Model	t_50_** (Hours)	t_90_*** (Hours)		
NPMBs	0.724292	0.82468	0.894398	1.17	3.19	0.403	92.7
PVDF	0.867891	0.9383	0.968293	16.7	31.9	0.307	14.4
PVDF/PVP	0.825071	0.906514	0.935472	10.7	19.8	0.575	19.44
PVDF/PVP 0.5 GO	0.973343	0.998133	0.998647	4.3	8.6	0.5	45.3
PVDF/PVP 0.5 GO/MOF	0.820906	0.903313	0.939609	3.19	6.65	0.45	53.53

n is the diffusion exponent, t_50_** is time required for 50% of the drug to be released, t_90_*** is time required for 90% drug release, ^‡^ RE_0–6 h_ is the release efficiency of the drug from 0 to 6 h, and ^†^ R^2^-value is the value of the regression co-efficient.

**Table 2:** Release kinetics parameters of donepezil loaded onto uncoated and polymer-coated alumina NBMBs. Stage 2.

**Formula Code**	**R^2^-Value ^†^**			**Korsmeyer–Peppas Model**		**n**	**RE_6–168 h_^‡^** **(%)**
	Zero-Order	Fickian Diffusion	Korsmeyer–Peppas Model	t_50_** (Hours)	t_90_*** (Hours)		
NPMBs	0.962	0.988	1	---	---	0.008	99.9
PVDF	0.998	0.998	0.984	30.1	43.3	0.18	24.2
PVDF/PVP	0.982	0.9981	0.9991	24	32.5	0.177	30.5
PVDF/PVP 0.5 GO	0.9022	0.948	0.979	14.4	11.8	0.104	60.95
PVDF/PVP 0.5 GO/MOF	0.99998	0.993	0.9660	14.4	10.2	0.0898	66

n is the diffusion exponent, t_50_** is time required for 50% of the drug to be released, t_90_*** is time required for 90% drug release, ^‡^ RE _6–168 h_ is the release efficiency of the drug from 0 to 6 h, and ^†^ R^2^-value is the value of the regression co-efficient.

## Data Availability

Data will be made available upon request.
